# CRISPR/dCas9-Based Systems: Mechanisms and Applications in Plant Sciences

**DOI:** 10.3390/plants10102055

**Published:** 2021-09-29

**Authors:** Chou Khai Soong Karlson, Siti Nurfadhlina Mohd-Noor, Nadja Nolte, Boon Chin Tan

**Affiliations:** 1 Center for Research in Biotechnology for Agriculture (CEBAR), Universiti Malaya, Kuala Lumpur 50603, Malaysia; karlson@um.edu.my; 2 Institute of Microengineering and Nanoelectronics (IMEN), Universiti Kebangsaan Malaysia, Bangi 43600, Malaysia; sitinurfadhlina@ukm.edu.my; 3 Department of Plant Sciences, Wageningen University & Research, Droevendaalsesteeg 4, 6708 PB Wageningen, The Netherlands; nadja.nolte@wur.nl

**Keywords:** CRISPR interference, CRISPR/dCas9 system, crop improvement, gene silencing, RNAi, transcriptional regulation

## Abstract

RNA-guided genomic transcriptional regulation tools, namely clustered regularly interspaced short palindromic repeats interference (CRISPRi) and CRISPR-mediated gene activation (CRISPRa), are a powerful technology for gene functional studies. Deriving from the CRISPR/Cas9 system, both systems consist of a catalytically dead Cas9 (dCas9), a transcriptional effector and a single guide RNA (sgRNA). This type of dCas9 is incapable to cleave DNA but retains its ability to specifically bind to DNA. The binding of the dCas9/sgRNA complex to a target gene results in transcriptional interference. The CRISPR/dCas9 system has been explored as a tool for transcriptional modulation and genome imaging. Despite its potential applications and benefits, the challenges and limitations faced by the CRISPR/dCas9 system include the off-target effects, protospacer adjacent motif (PAM) sequence requirements, efficient delivery methods and the CRISPR/dCas9-interfered crops being labeled as genetically modified organisms in several countries. This review highlights the progression of CRISPR/dCas9 technology as well as its applications and potential challenges in crop improvement.

## 1. Introduction

The recent advancement in omics-based technologies and bioinformatics methodologies has facilitated the ever-growing field of functional genomics and system biology [1,2]. With the help of such advanced technologies and gene regulating tools, such as RNA interference (RNAi), zinc finger nucleases (ZFNs) and transcription activator-like effector nucleases (TALENs), researchers are able to investigate the roles of specific genes in a cell or an organism. RNAi is a powerful method for gene function validation. This technology was first discovered in *Caenorhabditis elegans* [3,4]. RNAi is a conserved eukaryotic mechanism that uses small RNA molecules, such as small interfering RNAs (siRNAs) and microRNAs (miRNAs), to inhibit the translation of the target proteins. This method has been successfully applied in several crops to improve their resistance against pathogens like fungi, bacteria and insects [5] as well as abiotic stress tolerance, such as salinity and drought [6]. Despite its popularity, RNAi is hindered by several limitations, including inconsistency and incompleteness of knockdowns, potential non-specificity or off-target effects and inefficient delivery methods [7]. Genome editing tools, such as ZFNs and TALENs, seem a better alternative to RNAi since these techniques induce fewer off-target effects [8]. ZFNs and TALENs comprise a FokI nuclease domain and a DNA-binding domain. As the FokI nuclease domain requires dimerization to become active, a tail-to-tail orientation with appropriate spacing needs to be designed to enable dimerization of the FokI domains. This requirement provides specificity to ZFNs and TALENs. However, the synthesis of active nucleases is relatively expensive and difficult [9].

Deriving from the clustered regularly interspaced short palindromic repeats (CRISPR)/Cas9 system, the newly developed CRISPR/dCas9 has been repurposed for transcriptional regulation. This system consists of three major components: a catalytically inactive Cas9 (dCas9) protein, a customizable single guide RNA (sgRNA) that complementary to the promoter region of a gene and a transcriptional effector, either transcriptional activators (CRISPR activation; CRISPRa) or repressors (CRISPR interference; CRISPRi). The binding of dCas9/sgRNA and transcriptional effector complex to the promoter region of the downstream target genes results in transcriptional interference by blocking RNA polymerase binding or elongation. The CRISPRi functions analogously to RNAi in which both systems aim to silence or knockdown gene expression but possess different mechanisms and principles [8]. In essence, the CRISPRi method suppresses gene expression at a DNA level by preventing transcription, whereas RNAi uses a posttranscriptional mechanism by cleaving transcribed mRNAs.

CRISPR/dCas9 technology has revolutionized the fields of functional genomics. It is a simple, efficient and less expensive tool for targeted activation or repression of gene expression [10]. In this paper, we briefly discuss the discovery and principles of CRISPR as well as the development and recent progress of CRISPR/dCas9 technology. This review also highlights the applications and challenges of CRISPR/dCas9 in plant research.

## 2. The CRISPR/Cas System: Discovery and Principle

CRISPR was first discovered unintentionally in the genome of *Escherichia coli* by Ishino et al. [11] while sequencing the *iap* gene. The authors found an unusual series of tandemly repetitive 29 nucleotide (nt) DNA sequences interspaced with 32-nt spacer sequences downstream of the gene [11]. They have no clue about the biological function of these repeats since it lacks sequence homology to other known sequences at that time. Later, while sequencing numerous fragments in the genome of *Haloferax mediterranei*, Mojica et al. [12] discovered long tandem repeats. This marked the first time that direct repeats were detected in archaea. Mojica et al. [13] classified such interspaced repeat sequence as short regularly spaced repeats (SRSRs). To avoid naming confusion in future studies, Jansen et al. [14] together with Mojica and colleagues renamed these sequences as clustered regularly interspaced short palindromic repeats (CRISPRs). The CRISPRs-associated genes (Cas), *cas1* to *cas4*, were also identified in the same year by Jansen et al. [14].

In 2005, it was discovered that the spacers within CRISPRs were derived from invading phages and plasmids [15,16,17]. These findings provided a clue that CRISPR/Cas could be an adaptive immunity system in prokaryotes. The role of CRISPR/Cas systems as an adaptive immunity has later been supported by experimental findings from several research groups [18,19,20,21]. These authors found that new spacer sequences from the infecting phage are acquired into bacterial CRISPR array.

The CRISPR/Cas systems can be classified into three major types (Types I, II and III). Type I and III systems employ multi-Cas proteins for target recognition and cleavage. For example, Type I system uses Cas3 to cleave target DNA [22], whereas Type III system utilizes Cas10 with the aid of polymerase and repeat-associated mysterious proteins (RAMPs) to cleave RNA and DNA [23]. In comparison, Type II system only needs a single effector protein (Cas9) to accomplish the interference and, thus, is relatively simple to be engineered to function as a genome-editing tool. In the CRISPR/Cas systems, a trans-activating crRNA (tracrRNA) will bind to the repeat sequence of pre-crRNA to form mature crRNAs with the aid of Cas9 and endoribonuclease III (RNase III) [24]. The mature dual tracrRNA:crRNAs form a complex with a Cas9 protein that can recognize the protospacer adjacent motif (PAM) and cleave specifically at 3 bp before the PAM site of the double-stranded DNA [25]. In 2012, the research group led by Emmanuelle Charpentier and Jennifer A. Doudna published a landmark paper [26], detailing the application of class II CRISPR/Cas9 system for gene editing. This ground-breaking discovery has earned these two scientists the 2020 Nobel Prize in Chemistry.

## 3. The Current CRISPR/Cas9 System and Strategies to Mitigate Off-Target Effects

The newly developed CRISPR/Cas system replaces the dual-tracrRNA:crRNA with an artificial sgRNA which can be easily altered to complement the targeted DNA sequence (the region 20-nt upstream of the PAM site) to induce double-stranded breaks (DSBs) at the desired position [27]. The DSBs will then be repaired by nonhomologous end-joining (NHEJ) or homology-directed repair (HDR), resulting in the formation of insertions or deletions (indels) in coding regions [28]. CRISPR/Cas9 is now widely used to study gene function and develop genetically modified (GM) organisms.

Being an innovative and cutting-edge tool for gene editing, CRISPR/Cas9-based genome editing has been used to improve commercially important crops, including rice, wheat and cotton [29]. It can be used to introduce heritable trait-related mutations or knock out genes conferring undesirable traits. This approach has been used to increase tolerance to abiotic stress [30,31,32] and disease resistance [33,34,35]. For example, CRISPR/Cas9-based genome editing has been used to introduce indels affecting eukaryotic translation initiation factor 4E proteins to enhance resistance against multiple RNA viruses in cucumber [36]. In addition, targeting the coding region of a gene, this approach could be used to introduce indels in the promoter region. An example of this application was the use of CRISPR/Cas9 to alter the promoter of the rice sugar transporter gene *OsSWEET14*, which successfully induced resistance to bacterial blight [37]. CRISPR/Cas9-based genome editing could be used to enhance photosynthesis efficiency and yield in C3 plants, such as rice. Recently, Zheng et al. [38] developed rice expressing a Cas9 targeting the hexokinase gene *OsHXK1* and observed a significant increase in light saturation points, stomatal conductance, light tolerance, photosynthetic products and rice yields. Another application of CRISPR/Cas9-based genome editing is crop biofortification. Staple crops contribute calories, but they fail to meet the micronutrient demands. Hence, the biofortification of staple crops has been proposed as a strategy to ameliorate nutrient deficiencies. However, biofortification using conventional breeding is challenging because the changes required in the genome are too complex [39], which makes genome editing the possible approach. The use of CRISPR-mediated genome editing has been applied in several crops [40,41] and reviewed by Ahmad et al. [42].

Although the CRISPR/Cas9 system is an important tool for crop improvement, its high frequency of off-target activity compared to other engineered nucleases has become a major concern [43,44]. Many efforts have been made to improve the specificity of the CRISPR/Cas9 system for gene editing and the relevant approaches to reduce the off-target effects. These include:

(a) bioinformatics selection and modification of sgRNA;
(b) finetuning expression of CRISPR components;
(c) use of Cas9 variants and orthologs;
(d) utilization of heterologous nucleases in the CRISPR system;
(e) alternative CRISPR approaches.

### 3.1. Bioinformatics Selection and Modification of sgRNA

sgRNA is an important component in the CRISPR system. As it functions as a guide to Cas9, the design of sgRNA is crucial to reduce the off-target mutation. For instance, sgRNAs with high GC content (40–60%) have been shown to improve the on-target activities in wheat [45]. If the high percentage of GC is more proximal to the PAM site, the efficiency of on-target gene editing would be higher [46]. The length of sgRNA is another important aspect for the occurrence of unwanted mutations. Ran et al. [47] found that a shorter length of sgRNA (17 or 18 bp instead of 20 bp) exhibited a 500-fold decrease in off-target events while maintaining the on-target accuracy. A recent strategy utilizing the dead truncated sgRNA (dead RNA off-target suppression (dOTS)) has been shown to reduce off-target effects and increase the on-target activity by 40-fold [48]. sgRNAs can also be modified chemically by incorporating substances, such as 2′-*O*-methyl-3′-phosphonoacetate, in the sgRNA ribose-phosphate backbone to mitigate the off-target effects [49]. Through this modification, the off-target cleavage was significantly reduced up to 120-fold while maintaining its on-target performance [49]. Other modifications include the partial substitution of crRNAs with DNA [50], thiophosphonoacetate linkages at the termini [51] or internal residues [52], site-specific incorporation of 2′-4′ bridged nucleic acids [53], as well as 2′-*O*-methyl, 2′-4′ bridged nucleic acid and phosphorothioate linkages [54].

### 3.2. Finetuning Expression of CRISPR Components

In cells, the expression of CRISPR components, such as Cas9 and sgRNA, is vital to control the off-target effects. The specificity and activity of the Cas9/sgRNA complex are often highly condition-dependant. A lower concentration of Cas9/sgRNA complex in cells will reduce the probability of off-target effect, although there might be a trade-off for decreased efficiency at the on-target site. By titrating down the amount of Cas9 and sgRNA expression plasmid in transfecting cells, Hsu et al. [55] successfully reduced the off-target effect, while maintaining the on-target efficiency [55].

Rapid degradation of the CRISPR components in cells may also decrease the off-target effects [56]. The prolonged incubation period of the CRISPR components in cells might increase the risk of off-target binding and cleavage. Given that most CRISPR components are delivered by either plasmid transfection or viral vector integration, an alternative direct delivery method has been developed to shorten the exposure duration of the Cas9/sgRNA complex in cells. For instance, direct delivery of the Cas9 protein and *in vitro* transcribed sgRNA, either individually or as purified complex (ribonucleoproteins; RNPs), reduced off-targets in cells [56]. Kim et al. [57] demonstrated that the RNPs were immediately degraded after targeting the *CCR5* gene, generating fewer off-target mutations compared to the plasmid transfection [57].

### 3.3. Use of Cas9 Variants and Orthologues

The requirement of a PAM sequence of 5′-NGG-3′ has restrained the Cas9 targeting range. Given that different bacterial strains contain Cas9 proteins recognizing different target PAM sequences, the use of Cas9 orthologs from other bacteria and variants can overcome this limitation (reviewed by Gasiunas et al. [58]). The Cas9 proteins obtained from *Staphylococcus aureus* (SaCas9) and *Neisseria meningitidis* (NmeCas9) were found to recognize the PAM sequence of 5′-NNGRRT [59] and 5′-NNNNGATT [60], respectively. The introduction of Cas9 orthologs in an organism may not interfere with Cas9. As shown by Steinert et al. [61], SaCas9 and Cas9 did not interfere with each other, indicating the possibility of editing target regions using different Cas9 orthologs. The use of the NmeCas9 ortholog has significantly reduced the off-target cleavage and increased the target specificity in mammalian cells by exhibiting lower tolerance to base mismatches and DNA bulges [60]. Müller et al. [62] edited the *PRKDC* and *CARD11* genes using Cas9 cassettes isolated from *S. thermophilus* (St1Cas9 and St3Cas9), where the authors found that only a few to no off-target effects have been detected. Nishimasu et al. [63] developed an engineered Cas9, *Streptococcus pyogenes* Cas9 (SpCas9)-NG, a variant of Cas9 that can recognize NG-PAM instead of NGG-PAM, to expand the targeting range and improve its compatibility to the target genomic loci. This approach has also been used to edit genes in *Arabidopsis* [64] and rice [65].

Other modified Cas9 proteins, such as enhanced-specificity eSpCas9 variant [66], hyper-accurate Cas9 variant, HypaCas9 [67] and high-fidelity SpCas9-HF1 [68], have also been reported. These modified Cas9 proteins were shown to nearly entirely avoid nonspecific DNA editing. Zhang et al. [69] demonstrated that the on-target:off-target indel frequency ratio for eSpCas9 and SpCas9-HF1 was 273-fold higher than the wild type SpCas9, showing its high efficiency in gene editing. The recent discovery of the smallest Cas9 ortholog, *Campylobacter jejuni* CAS9 (CjCas9), has also greatly improved the off-target effect without comprising its on-target activity [70].

### 3.4. Utilization of Heterologous Nucleases

The structure of the Cas9 has been modified to reduce the off-target effect. For instance, the D10A Cas9 nickase (nCas9), an example of Cas9 mutants, has been shown to have a lower off-target rate because of the structural changes in their binding region [71]. Instead of directly inducing DSB, nCas9 only produces a nick or single-stranded break at the target site. The paired binding of the nCas9 on the opposite strand produces DSB at a higher specificity with reduced potential off-targets by doubling the recognition site of the target gene [72]. This paired nicking strategy could generate 5′ overhangs and spur the formation of indels more frequently [73,74]. Fusing the FokI nuclease domain to either dCas9 [75] or nCas9 [76] is another strategy to increase the specificity of gene targeting and reduce the off-target effects. This RNA-guided FokI-Cas9 nuclease requires dimerization, similarly to the nCas9. This approach has been shown to decrease the off-target activities by 40% compared to Cas9 [77].

### 3.5. Alternative CRISPR Approaches

In addition, modifying the nCas9, a new technique called base editing has also been developed. This approach allows direct conversion of one target DNA base into another base without DSBs [78]. By fusing nCas9 with adenine base editors, CRISPR-mediated base editing enables the conversion of A-T to G-C, while fusion with cytosine base editors can alter an A-T base pair into a C-C base pair [79,80]. Shimatani et al. [81] successfully developed herbicide-resistant rice plants using a base editing approach through a C287T mutation on acetolactate synthase. The C287T mutation leading to an A96V amino acid substitution endows rice plants resistant to herbicide imazamox. Despite its great efficiency in editing the DNA, the ability of CRISPR/Cas9 DNA base-editing technology to generate precise base-edits beyond the four transition mutations is still a major limitation.

Prime editing, another recent DSB-free method, has been developed to overcome these shortcomings. This method employs an engineered reverse transcriptase fused to nCas9 and a prime editing guide RNA (pegRNA) [82]. The pegRNA differs from sgRNAs as it consists not only the guide sequence that can recognize the target sites but also a reverse transcriptase template spelling the desired genetic changes. Lin et al. [83] recently adapted prime editors to induce point mutations, insertions and deletions in rice. Through this approach, all twelve kinds of base-to-base substitutions, as well as multiple base substitutions, insertions and deletions, were detected. The authors reported that the frequency of prime editing induced by this prime editor was up to 21.8% [83]. Similar findings have also been reported by other researchers [84,85]. Although prime editing has several advantages compared with other techniques, including enabling precise sequence deletion, addition and substitution, this technique is still in its infancy. The specificity and potential for off-target modifications of this technology have yet to be investigated.

CRISPR-based epigenetic engineering could be used to target epigenetic factors, such as histones and methyltransferases. Combining the epigenetic modulators with the dCas9 could characterize and map the chromatin marks in the DNA region. The recent CRISPR technologies for epigenome editing have been summarized by Nakamura et al. [86]. However, this approach is also prone to some levels of off-target activity [87]. The off-target methylation might be due to the DNA methyltransferase activity of the fusion protein. Stepper et al. [88] showed that by reducing the DNA methyltransferase multimerization and lowering the catalytic activity of the fusion protein, a lower off-target effect could be obtained.

## 4. Inactive CRISPR-Associated Nucleases: A Transcriptional Regulator

**Figure 1:**
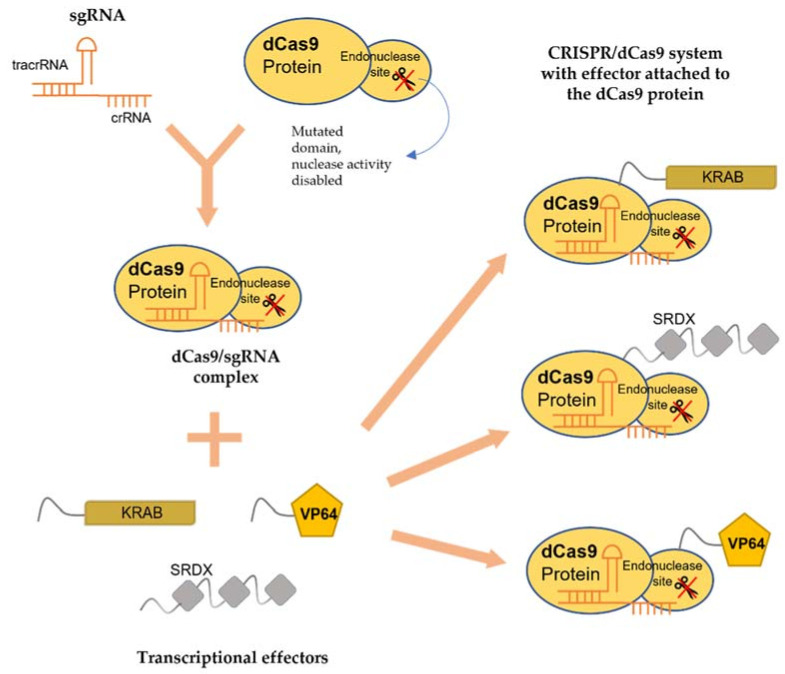
Schematic illustration showing sgRNA, dCas9 and transcriptional effectors. dCas9 together with sgRNA forms the dCas9/sgRNA complex for transcriptional regulator attachment. Fusion of the complex with effectors, transcription activators or repressors, are used for targeted gene regulation.

**Figure 2:**
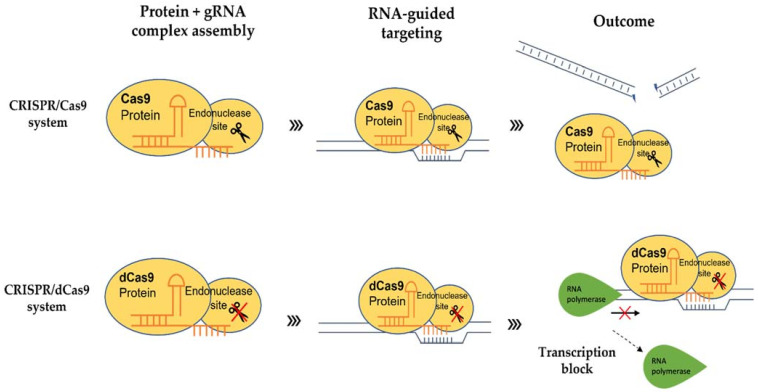
Schematic diagram showing the difference between the CRISPR/Cas9 system and the CRISPR/dCas9 system utilizing Cas9 and deactivated Cas9 (dCas9), respectively.

In addition, being a molecular scissor, CRISPR technology has been developed to be a sequence-specific and non-mutagenic gene regulation tool to regulate both transcriptional and epigenetic processes. The use of CRISPR/dCas9 technology was first reported by Qi and colleagues in 2013 [89]. This technology exploits the deactivated variants of the Cas9 enzyme (dCas9), guided by a sgRNA forming a dCas9/sgRNA complex, that is incapable of cleaving DNA but retains its ability to specifically bind to the DNA (Figure 1). By targeting gene at the promoter or coding sequence, the complex pairing with a transcriptional effector, either repressor or activator, interferes with the binding of RNA polymerase (RNAP) (Figure 2). Without the binding of RNA polymerase and transcription factors, the transcription of the target gene is inhibited [90]. dCas9 can also be fused to an epigenetic modulator, such as methylation enzyme, to generate dCas9-tethered epigenetic enzymes for targeted regulation at defined genomic loci [91]. The CRISPR/dCas9 system contains three main components: (i) sgRNA, (ii) dCas9 and (iii) the transcriptional effector, which will be discussed below.

### 4.1. sgRNA

sgRNA is a combination of crRNA and tracrRNA where they can be linked together with a loop sequence to form the chimeric sgRNA [92] (Figure 1). The structure of sgRNA in CRISPR/dCas9 is largely the same as in CRISPR/Cas9 system. The two major regions of the sgRNA, i.e., (i) the spacer and (ii) scaffold regions, are of particular importance in the CRISPR system since both regions will form a complex with dCas9 to direct targeted transcriptional regulation. The spacer region contains the crRNA, a 17–20 nt sequence complementary to the promoter region of a target gene, while the scaffold region contains the tracrRNA, which acts as the binding scaffold to bind to the dCas9 protein. By choosing appropriate sgRNAs, the dCas9/sgRNA complexes can be guided to bind to the target gene. To increase the efficiency of CRISPR/dCas9 in transcriptional regulation, the position of the target region is vital. For activation, sgRNA is often designed to target −400 to −50 bp upstream of the translation start site (TSS), while −50 to +300 bp at the TSS is commonly used for repression [93]. The easy modification of the spacer region and the synthesis of sgRNAs have made CRISPR/dCas9 becomes a powerful tool in regulating the expression of transcript levels in *planta*.

### 4.2. dCas9

dCas9 is a mutated wild type Cas9 (sometimes referred to as dCas9 null mutant). The nuclease domains of Cas9 have been altered by mutating H840A in the HNH domain and D10A in the RuVC1 domain [89] (Figure 1). The dCas9 is incapable of cleaving DNA but is still able to bind to the target genes with the same specificity when guided by sgRNA. Using dCas9 alone, the transcript level of endogenous *TEF1* in *Saccharomyces cerevisiae* was repressed up to 18-fold [93]. The silencing of the dCas9 is reversible, which means dCas9 can regulate the expression of a gene without modifying the genome permanently. Li et al. [94] demonstrated that *in vitro* silencing of *yfp* gene by dCas9/sgRNAs under an arabinose-inducible promoter could be reversed by removing the inducer, arabinose.

### 4.3. Transcriptional Effectors

Transcriptional effectors are chimeric proteins that contain DNA-binding domains [95]. These effectors can be fused with dCas9 protein to modulate gene expression. If the CRISPR/dCas9 system is paired with a synthetic transcriptional repressor, the expression of the target gene will be repressed (CRISPRi) (Figure 1). One such example is Kruppel-associated Box (KRAB) [96], which has been commonly used for dCas9-based repression studies [97]. Piatek et al. [95] reported that the transcription activity of *PDS* was remarkably reduced by dCas9:SRDX (the combination of SRDX effector with dCas9 for repression) compared to control (dCas9 alone). On the other hand, if the CRISPR/dCas9 system is fused with a synthetic activator effector like Herpes simplex viral protein (VP16), transactivation domains of zinc-finger proteins, or transcription activator-like effector (TALE), the transcription of the target gene can be activated (CRISPRa) [98]. To modulate epigenetic marks, different histone post-translational effectors and domains are fused to dCas9 [99]. These include DNA methyltransferases, ubiquitin ligases and methylcytosine deoxygenases [100,101].

## 5. Strategies for Programable Transcriptional Regulations in Plants

RNA-guided transcriptional regulation of a gene is a complex process. This process involves the recruitment of activating and repressing transcription factors that are spreading across a large region of the genome [102]. The binding of these regulators to their target DNA sequences can be hampered by epigenetic modifications like histone acetylation and DNA methylation [103]. DNA methylation has been shown to disrupt 76% of transcription factors from binding to their target DNA sequences in *Arabidopsis*, indicating that epigenetic modifications could affect the transcription state of genes [104]. The efficiency of repression is also dependent on the host systems. When comparing prokaryotic and eukaryotic cells, the efficiency for dCas9 to repress the expression of monomeric red fluorescent protein (*mRFP*) in eukaryotes was much lower (20-fold) [93] than prokaryotes (up to 1000-fold) [89,105]. Hence, multiple strategies to overcome the low efficiency of transcriptional regulations in eukaryotic cells are indispensable.

### 5.1. Multiple sgRNAs

**Figure 3:**
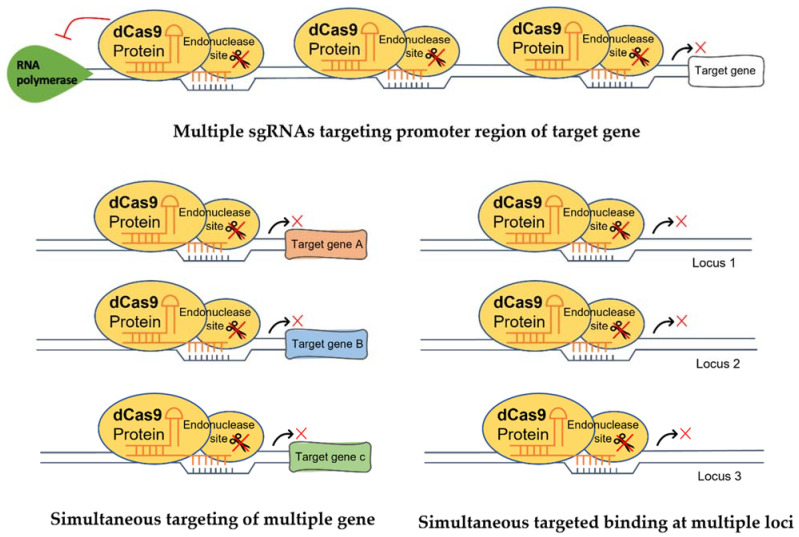
A schematic diagram shows the multiplexability of the CRISPR/dCas9 system. Enhancement of transcriptional regulation, simultaneous targeting of different genes and simultaneous targeted binding at multiple loci are possible using multiple sgRNAs.

sgRNAs can be easily manipulated to target several regions of a gene simultaneously. This strategy allows dCas9 to be guided by multiple sgRNAs to bind to different target loci simultaneously [106]. When combining two sgRNAs (each sgRNA has 300-fold repression), Qi et al. [89] found that the expression of the *mRFP* gene was repressed up to 1000-fold. The authors also found that the combination of two weaker sgRNAs (each with only 5-fold repression) produced a multiplicative suppression effect up to 20-fold in *E. coli*. This strategy has also been used in plants. In maize, a combination of 2 sgRNAs with a suppressor dCas9 to target the promoter region of maize *phytoene desaturase1* (*PDS1*) showed a 60% reduction of *PDS1* expression, whereas a 2.5-fold increase of *PDS1* expression was detected when using an activator dCas9 [107]. Li et al. [108] reported that the expression of multiple endogenous genes, *WRKY30*, *RLP23* and *CDG1*, was enhanced using three sgRNAs. Taken together, these studies showed that multiple sgRNAs could efficiently regulate the expression of a target gene at several regions simultaneously (Figure 3). This is particularly useful for targeting groups of genes in metabolite biosynthetic pathways to enhance the production of desired metabolites. Despite its effectiveness in regulating gene transcription, precautions should be taken to avoid both sgRNAs to compete for the same region.

### 5.2. Modification of CRISPR/dCas9 Components

**Figure 4:**
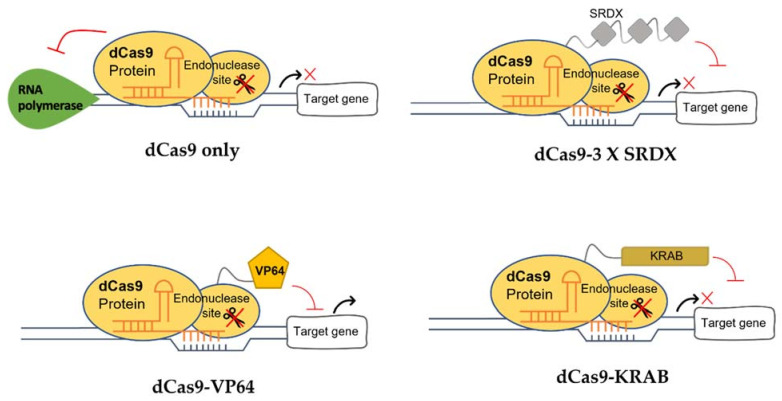
Different types of dCas9 protein with or without the effectors, such as KRAB and VP64.

The CRISPR/dCas9 components, i.e., dCas9 and effectors (such as KRAB (repression) and VP64 (activation) (Figure 4)), can be modified to enhance the efficiency of transcriptional regulation. The different designations and modifications of CRISPR components for transcriptional regulation are summarized in Table 1.

In CRISPRi, the binding of dCas9 at the target site of a gene prevents the transcription from initiating. Although dCas9 alone can interfere with the transcriptional machinery, its repression level is influenced by different repressors. A modified dCas9, dCas12a (also known as dCpf1), has been found to show better transcriptional repression compared to dCas9 [116]. dCas12a can process a single transcript tandem crRNA array to multiple crRNAs on its own, enabling a more simplified multiplex transcriptional repression compared to dCas9 [117].

CRISPR/dCas9 could also be used for gene activation (CRISPRa), depending on the effector attached to the dCas9. For example, when the dCas9-VP64, directed by sgRNA, binds to the promoter of a target gene, the complex can recruit transcription factors and subsequently regulate transcription of the gene. Many other activation effectors have been developed. Some strategies to achieve better transcriptional activation of a gene are discussed below.

Tethering activators through protein-recruiting system can also enhance the plant transcriptional activation. Tanenbaum et al. [118] established a dCas9-SunTag system that could improve endogenous gene expression. In this system, an activator-recruiting scaffold in the effector has been modified by fusing dCas9 with a tandem array of peptides, known as the SunTag array. This protein scaffold (repeating peptide array) can recruit multiple antibody-fusion proteins. For instance, Tanenbaum et al. [118] fused a dCas9 with a general control protein 4 (GCN4) peptide array, which can recruit multiple copies of single-chain variable fragment-superfolded green fluorescent protein (sfGFP)-synthetic transcriptional activator VP64 (scFV–sfGFP–VP64) GCN4 antibody to a single dCas9. Through this strategy, Tanenbaum et al. [118] was able to enhance the activation efficiency of the *CXCR4* and *CDKN1B* genes by recruiting many copies of the VP64 effectors instead of one VP64. Recently, Papikian et al. [114] examined the effect of *FWA* gene in *Arabidopsis* flowering by activating this gene using the dCas9-SunTag system. They found that the methylated and silent *FWA* can be upregulated by the dCas9-SunTag system and the effect could be detected up to T2 generation.

The recently developed synergistic activator mediator system (SAM), where an additional sgRNA is engineered through the attachment of a minimal hairpin aptamer to the tetraloop and stem loop 2 of sgRNA, could increase the transcriptional activation. This aptamer is able to bind to the dimerized MS2 bacteriophage coat proteins, forming the MS2-mediated sgRNA (msgRNA). After fusing with activators, such as p65 and HSF1 transactivation domains, this SAM complex will increase the recruitment of transcription factors and subsequently activate the endogenous gene expression up to 105-fold [119]. When the SAM strategy was introduced into *Arabidopsis* plants, Park et al. [120] could trigger the transcriptional activation of the endogenous Anthocyanin pigment 1 (*PAP1*) and vacuolar H+-pyrophosphatase (*AVP1*) genes by 2- to 5-fold, respectively. The low transcripts of the target genes might be due to the native regulatory repression factor in the promoter [121].

CRISPR-Act2.0, a new strategy that similar to the design of the SAM, has been recently developed in plants. This strategy uses dCas9-VP64 together with a modified sgRNA which consists of two internal MS2 RNA hairpins. These MS2 RNA hairpins can facilitate the recruitment of additional VP64 via MCPs [122]. It has been shown that the CRISPR-Act2.0 system was better than dCas9-VP64 in activating both protein-coding and non-coding genes in *Arabidopsis* and rice [113].

### 5.3. Transcriptional Regulation Toolbox

The construction of CRISPR/dCas9 to efficiently target multiple genomic loci poses a significant challenge. The sequences of the designed sgRNAs and dCas9-effector are often placed in a single T-DNA region [123]. This requires highly specialized skills. Without experience, cloning such constructs could be time-consuming and laborious. To address this shortcoming, a streamlined toolbox utilizing the recent cloning methods, such as Golden Gate and Gateway assembly, has been developed. Using this strategy, a pro-dCas9-3X (SRDX) repressor can be easily constructed in 10 days [110]. Lowder et al. [110] found that the expression of *AtCSTF64* in *Arabidopsis* was repressed by 60% using this construct. Another two T-DNA constructs and three sgRNAs were also developed together with this toolbox to target *PAP1* and miR319. Lowder et al. [110] found an increase of 2- to 7-fold and 3- to 7.5-fold of *AtPAP1* and miR319, respectively, was recorded in the transformed *Arabidopsis* compared to control. Such targeted gene regulation is expected to allow robust multiplexing in the plant genome.

### 5.4. Plant Specific Transcriptional Effectors

Plant transcriptional effectors, such as ethylene response factors (ERFs), are an important modulator for gene expression. These transcription factors have been evaluated for developing dCas9-based gene activators in plants. ERFs are important ethylene-signalling regulators for plant defence response against abiotic and biotic stresses. Using CRISPR/dCas9 system, the sgRNAs can be designed to target the region upstream of the TATA box and TSS of ERF gene for gene activation.

Among the ERFs, the ERF/EREBP family is particularly crucial as these regulators contain domains with motifs that are unspecified to DNA binding [124]. In the ERF/EREBP family, the SRDX derived from the ERF-related amphiphilic repressor domain (EAR) was found to confer repression activities [125]. Fusing of the EAR domain to dCas9 has been used to target the Bs3::uidA [95] and *PDS* genes [126] in tobacco. Another ERF transcriptional regulator, the EDLL motif, which is a strong activation domain has also been used to activate several genes, such as *PAP1* and *FIS2* in *Arabidopsis* [113,123]. However, the dCas9-EDLL with a single sgRNA only showed modest transcriptional activation activities in plant cells [109]. Although an attempt to fuse the EDLL motif with VP64 to boost the efficiency of transcriptional activation, it failed to work in plant cells [113].

## 6. Application of CRISPR/dCas9 in Plants

The CRISPR/dCas9 system has emerged as one of the most efficient and cost-saving tools in molecular biology. In addition, studying gene function, the CRISPR/dCas9 can also be applied for plant improvements, such as improving resistance/tolerance of plants against biotic and abiotic stresses, regulation of secondary metabolites and cell imaging (Table 2).

### 6.1. Enhancing Abiotic Stress Tolerances in Plants

Abiotic stresses, such as drought, flooding, salinity, heavy metals and heat, have adversely affected the growth and fitness of the plants. Despite extensive research efforts, a feasible and effective method to enhance abiotic stress tolerance in plants is still lacking. This might be due to the complex regulatory networks, including multifaceted interactions between metabolic, signalling and regulatory pathways, in plants [133]. The use of CRISPR/dCas9 could be beneficial in improving plant stress tolerance (Table 3). To enhance drought tolerance of *Arabidopsis*, Paixão et al. [134] introduced a construct, where the dCas9 fused with the Arabidopsis histone acetyltransferase 1 (AtHAT1), to activate the abscisic acid (ABA)-responsive element binding protein 1/ABRE binding factor (AREB1/ABF2). The authors observed that the drought-stressed transgenic plants have a higher survival rate and chlorophyll content than control. Recently, de Melo et al. [135] reported that *AREB-1*-activated *Arabidopsis* by CRISPRa showed an improved drought tolerance compared with wild type plants. A 2-fold higher relative water content and lower level of malonaldehyde were observed in those transgenic *Arabidopsis* [135]. Park et al. [120] found that a higher accumulation of K^+^ and Na^+^ ions was detected in transgenic *Arabidopsis* with 2- to 5-fold higher *AVP1* expression and improved tolerance to drought stress compared with wild type after activating the transcription of *AVP1* using a redesigned CRISPR/dCas9 activation system. They redesigned their CRISPR/dCas9 activation system by adding a heat-shock factor 1 activation domain and the p65 transactivating subunit of NF-kappa B to the dCas9-VP16.

### 6.2. Improving Plant Immunity against RNA Virus

Viruses may affect the growth of their plant hosts, causing a significant loss for the agricultural sectors [136]. Viruses incorporate their genetic material into the plant genomes to reproduce and fabricate the building blocks for new virus particles. Plants defence themselves against virus invasion by activating their RNAi machinery. However, many viruses could inhibit the plant RNAi silencing pathway by releasing a suppressor protein to prevent siRNAs from initiating the process [137]. Since the CRISPR/dCas9 does not have the same silencing pathway as the RNAi, it is more desirable to use such technology to target the viral RNA and disrupt their invasion. Several recent studies have been carried out to explore the feasibility of CRISPR/dCas9 in improving plant immunity after the reports on inhibiting virus *in vivo* using variants of Cas protein, namely Cas9 from *Francisella novicida* (FnCas9) and the Cas effector from *Leptotrichia shahii* (LshCas13a) or *Leptotrichia wadei* (LwaCas13a) [138,139]. Zhang et al. [140] reported a 40–80% reduction of cucumber mosaic virus (CMV) and tobacco mosaic virus (TMV) accumulation in *N. benthamiana* and *Arabidopsis* using FnCas9 and discovered that FnCas9 inhibits the virus in a CRISPRi fashion. The repression of CMV virus was not affected even without the endonuclease’s activity of FnCas9, indicating that the RNA-virus inhibition by FnCas9 is due to its RNA-binding capability. As demonstrated by Zhang et al. [140], this CRISPR/dCas9 system could be potentially used to develop stable transgenic RNA-virus resistant plants since the resistance against CMV in *Arabidopsis* can be detected up to T6 generation. Another study by Khan et al. [141] showed that the accumulation of Cotton Leaf Curl Virus (CLCuV) in tobacco was decreased by 60% using CRISPR/dCas9 compared to control. It is noteworthy that the efficiency of CRISPR/dCas9 was found to be lower than TALE (80%) in inhibiting CLCuV replication. This might be because TALEs are a natural transcription factor that are well adapted in plants [141]. However, the multiplexability and the ease of designing sgRNAs in the CRISPR/dCas9 system is still an alternative for the inhibition of viral RNA. To reduce turnip mosaic virus (TuMV) in tobacco, Aman et al. [142] developed a CRISPR/dCas9 construct containing Cas13a, which could process pre-crRNA into functional crRNA innately, to target the viral mRNAs. A recombinant TuMV expressing GFP (TuMV-GFP) was then agro-infiltrated into tobacco plants. The authors found that the intensity of GFP-expressing TuMV in tobacco was reduced up to 50%, indicating the successful control over the spread of the viral GFP signal.

### 6.3. Regulation of Secondary Metabolites

Plant secondary metabolites are important for plant growth and development. These metabolites have been extensively studied due to their medicinal properties [143]. To enhance the production of these useful metabolites, several strategies, such as conventional plant breeding and genetic engineering, have been adopted. Plant breeding, however, is a laborious and time-consuming approach as it involves lengthy crossing and backcrossing steps [144]. On the other hand, manipulation of secondary metabolite biosynthetic pathways at the molecular level has shown promising results but often requires the regulation of multiple key genes simultaneously. The common strategies for secondary metabolite enhancement are: (1) overexpressing key genes to ensure sufficient supply of precursors and increase metabolic flux through the target pathway; (2) silencing the key enzyme genes in the competitive pathway of the target metabolite to avoid intermediates being diverted; and (3) overexpressing transcription factors for activation or repression of multiple endogenous key genes simultaneously to enhance the synthesis of the metabolites. The biosynthesis of secondary metabolites is a complex process and often requires simultaneous expression of multiple genes. Multiplexed CRISPR/dCas9 technologies, in which a few sgRNAs or Cas proteins are expressed at once, could be a solution for this. For example, Reis et al. [145] recently reported that the amount of succinic acid in the CRISPRa-interfered bacteria was about 150-fold higher than control. They activated 6 succinic acid related genes by introducing 20 sgRNAs. To date, there are many reports on using CRISPR/dCas9 to enhance metabolite production in microorganisms [146,147,148,149]. However, to our knowledge, the use of CRISPR/dCas9 for plant secondary metabolite regulation has not been reported yet probably due to the complexity of plant secondary metabolisms and inefficient delivery methods.

### 6.4. Other Applications of CRISPR/dCas9

Genome structure is crucially important to the regulation of basic cell functions, such as accurate chromosomal separation in cell division, repair and replication in DNA, as well as gene expression [150]. To monitor these changes, fluorescent *in situ* hybridization (FISH) is often used. However, this technique requires one to sacrifice the precious samples as it involves cell fixation and DNA denaturation steps. On this basis, imaging-based CRISPR/dCas9 could serve as an alternative to the FISH method. A CRISPR/dCas9-based cell imaging technique has been developed by Dreissig et al. [127] through the fusion of two dCas9 orthologs (Sp-dCas9 and Sa-dCas9) with copies of fluorescence proteins to visualize telomeres and to view multiple genomic loci simultaneously in tobacco leaf cells. The authors showed that telomeres are localized in the periphery of interphase nuclei. However, in comparison with FISH, the efficiency of a telomere labeling by dCas9 was 70% [129]. To improve the labeling efficiency of CRISPR/dCas9 system, various orthologues of Sp-Cas9, including St1-Cas9 and Sa-Cas9, can be recruited in combination with modified sgRNAs with an RNA aptamer MS2/PP7 insertions that bind to a fluorescent coat protein [129]. Using this method, the dynamics of telomeres and centromeres in living plant cells can be traced.

The epigenetic regulatory mechanisms are essential for plant development and adaptation to the environment. As previously mentioned, dCas9 can be fused with epigenetic regulatory factors to modulate chromatin modifications. This makes the CRISPR-based epigenetic regulators a promising tool to investigate the relationships between specific phenotypes and chromatin features. However, the current approaches for the studies of epigenetic regulation are often tedious and costly since these techniques require intensive labor work and pose a risk of unspecific targeting. Since dCas9 can be fused with DNA methylase or demethylase to regulate the level of DNA methylation, the CRISPR/dCas9 technology could be used to understand epigenetic regulation. For example, dCas9 fused with mammalian acetyltransferase (p300) was used to target the promoter region of *IL1RN*, *MYOD1* (*MYOD*) and *POU5F1/OCT4* (*OCT4*) genes to enhance the histone H3 acetylation at lysine 27 [151]. Lee et al. [121] developed a CRISPR/dCas9 construct containing MS2 epigenetic regulator (dCas9-MS2VP64) to target the flowering time regulator *FT* gene in *Arabidopsis*. They found that about 65% of CRISPRa-interfered *Arabidopsis* showed a moderate shift in flowering time compared to the wild type [121]. Although most examples described here were developed in model species, we envisage that epigenetic versions of well-established alleles conferring favorable traits will be established in crop species.

## 7. Challenges and Issues of CRISPR/dCas9

Although there is the excitement of using CRISPR/dCas9 to facilitate advanced crop improvement, there are some challenges. Deriving from the CRISPR system, CRISPR/dCas9 shares the same limitations with the CRISPR system, i.e., off-target effects. While most off-targets occur when similar sequences are homologous to the desired sequence, these effects can also occur at proximal target regions with unrelated sequences [152]. To reduce the possibility of off-target effects, several free online prediction tools have been developed to assist researchers in designing sgRNA. These online tools are CRISPOR [153] and CCTop [154]. Another strategy by changing the structure of Cas9 to decrease its ability to bind to partly mismatched gRNAs could probably reduce the off-target effects. However, this strategy may have little significance for the CRISPR/dCas9 since the frequency of off-target effects for this technique is lower than the conventional CRISPR. Moreover, the off-target repression in CRISPR/dCas9 is reversible as it does not alter the genome sequence.

dCas9 binds to the promoter of target genes with the aid of sgRNA. However, a mismatch of one base pair in CRISPR/dCas9′s sgRNA decreases its performance, whereas multiple mismatches could make it inactive [94]. These mismatches could affect the binding activities of dCas9, preventing the CRISPR/dCas9 system from functioning correctly in cells [155]. In comparison, the CRISPR/Cas9 system edits DNA sequences regardless of the location in the genome, which can produce a higher frequency of off-target effects than CRISPR/dCas9.

Another challenge faced by CRISPR/dCas9 is the requirement of PAM sequences for the dCas9 to recognize the target gene. The PAM sequence determines the specificity of the CRISPR/dCas9 system. However, it may restrict the application of CRISPR/dCas9 if there are limited PAM sites in a genome. This limitation is same with the CRISPR/Cas9 genome editing. As described above, the recent discovery of Cas proteins that can recognize different PAM sequences could certainly help to expand the versatility of the CRISPR/dCas9 technology. Since CRISPR/dCas9 has lesser off-target effects and higher on-target efficiency than RNAi [156], it is probably a better alternative tool for gene functional studies. The RNAi approach is better suitable for high-throughput screening since less information is needed for the siRNA design.

Another limitation of CRISPR/dCas9 is that this system does not exist naturally in plants, meaning that CRISPR/dCas9 components like Cas proteins must be introduced into plant cells. The introduction of these components can be time-consuming [8] and sometimes requires codon optimization steps if the dCas9 is from different origins [157]. The current CRISPR/dCas9 delivery methods include *Agrobacterium*-mediated, polyethylene glycol (PEG)-mediated transformation and biolistic transformation or particle bombardment techniques. *Agrobacterium*-mediated transformation is the most widely used method due to its efficiency and relatively low cost. However, the requirement of a binary vector and the incorporation of a transgene in the plant genome is a major drawback. The PEG-mediated transformation involves laborious protoplast preparation. The particle bombardment method does not require isolation and culture of protoplasts and binary vectors containing a T-DNA, but its DNA fragment integration is random. The inefficient delivery of CRISPR/dCas9, the recalcitrance of plant tissues/cells and the inability of plant tissues/cells to regenerate into the plants remain as major barriers to realizing the potential of this technology. Furthermore, unintended genetic changes due to the nature of CRISPR activities might happen in the transformed plants [158,159]. Hence, a novel delivery method like a direct delivery of CRISPR/dCas9 constructs into plant apical meristem to circumvent tissue culture and a comprehensive risk assessment to evaluate the unintended changes are desirable.

The adoption of GM plants is highly affected by the regulations and society’s perception [160]. For example, only 11.9% of the population in China had a positive view on GM foods, whereas 41.4 and 46.7% had neutral and negative views, respectively [161]. Similar to the CRISPR-edited plants, each country has its perspectives on whether CRISPR/dCas9 like CRISPRi should be equally treated as traditional GM plants. The Chinese and EU governments have concluded that every organism “whose genome constitution has been changed by using genetic engineering technology” [162], to be applicable for CRISPR, are a GMO that needs strong regulation and pre-release authorization [163]. In the United States, if the CRISPR system is removed from the modified organism after the editing is executed, they may be classified as non-regulatory status [164]. For instance, a CRISPR-edited mushroom, modified to resist browning, was granted the non-regulatory status since no foreign DNA was detected [165]. To date, more than 100 GM plants have received the non-regulated status in the United States [166]. However, the CRISPR/dCas9-interfered plants may be regulated under the biosafety framework for GM plants since they contain a foreign dCas9. Hence, a regulatory risk assessment is essential to clarify if the consumption of CRISPR-interfered crops/fruits containing dCas9 could be harmful to humans. Conversely, such assessment may be different for CRISPR-plants with epigenetic modification if the final products are void of the CRISPR cassette.

## 8. Conclusions

CRISPR/dCas9 is an innovative and emerging technology for functional genomics. It could be used to regulate the transcription of targeted genes without altering their sequence. The CRISPR/dCas9 has now been used to enhance the resistance or tolerance of plants to biotic and abiotic stress and track the chromatin dynamics in live cells. With an improved CRISPR/dCas9 system through modifications of dCas9 proteins and effectors as well as the use of multiple sgRNAs or transcriptional regulation toolbox, we envisage that this technology would have a wide area of application, including plant secondary metabolite biosynthesis regulation. Despite its importance, several limitations of the CRISPR/dCas9 have to be addressed to fully exploit this technology. The concern of the public for possible hazards due to the consumption of a CRISPR/dCas9-interfered crop demand further investigation. In addition, there is a need to re-examine the regulations of CRISPR/dCas9-interfered crops. While there is much to be explored, CRISPR/dCas9 is undeniably a powerful tool that will develop into a mature technology to support plant genome engineering requirements.

## Figures and Tables

**Table 1:** Modification of CRISPR/dCas9 components for gene regulation in plant cells.

CRISPR/dCas9	Modification	Plant Species	Target Gene	Repression (%)/Activation (Fold-Change)	References
Transcriptional suppression	*dCas9-SRDX*	Nicotiana benthamiana	pNOS::LUC reporter	33	[95]
	*dCas9-BRD*	*N. benthamiana*	pNOS::LUC reporter	60	[109]
	*dCas9-3xSRDX*	*Arabidopsis*	CFTS64	60	[110]
	*dCas9-TALE-SRDX*	*Arabidopsis*	RD29-LUC (1)	-	[111]
	*dLbCpf1-SRDX*	*Arabidopsis*	miR159B	90	[112]
	*dAsCpf1-SRDX*	*Arabidopsis*	miR159B	90	[112]
Transcriptional activation	dCas9VP64	*Oryza sativa*	Os03g01240	2.0	[113]
	dCas9-TV	*O. sativa*	OsER1	62.0	[108]
	dCas9VP64+MS2-VP64	*O. sativa*	Os04g39780	4.0	[113]
	dCas9-VP64	*Arabidopsis*	pWRKY::luciferase	6.7	[108]
	dCas9-MCP-TV	*Arabidopsis*	AtWRKY	11.7	[108]
	dCas9-SunTag	*Arabidopsis*	AtCLAVATA3	100.0	[114]
	dCas9:SunTag-EDLL	*N. benthamiana*	pNOS::luciferase	3.0	[115]
	dCas9-VP64	*N. benthamiana*	pNOS::luciferase	3.0	[95]
	dCas9-EDLL	*N. benthamiana*	NbPDS	3.4	[95]

**Table 2:** Application of CRISPR/dCas9 in plants.

Application	Plant Species	Modification	Reference
Live cell chromatin imaging	*N. benthamiana*	dCas9-eGFP	[127]
		dCas9-FP	[128]
		dCas9-MS2-mRuby2	[129]
Transcriptional regulation	*Arabidopsis*	dCas9-MCP-TV	[108]
		dCas9-3xSRDX	[110]
		dAsCpf1-SRDX	[112]
	*O. sativa*	LUC/dCas9-TV	[123]
		dCas9VP64+ MS2-VP64	[113]
		dCas9-TV-6 × His	[108]
	*N. benthamiana*	dCas9-VP128	[95]
		dCas9-EDLL	[95]
Epigenetic manipulation	*Arabidopsis*	dCas9-MS2	[121]
		dCas9-TET1cd	[130,131]
		dCas9-SunTag	[114]
Chromatin topology	*Arabidopsis*	dCas9-PYL1	[132]
		dCas9-ABI1	[89]

**Table 3:** Application of CRISPR/dCas9 on abiotic stresses in plants.

Abiotic Stress	Plant Species	Target Gene	Modification	Reference
Drought	*Arabidopsis*	*AREB1/ABF2*	dCas9-AtHAT1	[134]
	*Arabidopsis*	*AREB-1*	dCas9-HAT	[135]
	*Arabidopsis*	*AVP1*	dCas9-VP16-p65	[120]
